# The Pathogenesis of Sepsis and Potential Therapeutic Targets

**DOI:** 10.3390/ijms20215376

**Published:** 2019-10-29

**Authors:** Min Huang, Shaoli Cai, Jingqian Su

**Affiliations:** 1 Biomedical Research Center of South China, College of Life Science, Fujian Normal University, Fuzhou 350117, China; lll.suspendded@gmail.com; 2 The Key Laboratories of Innate Immune Biology of Fujian Province, Fujian Normal University, Fuzhou 350117, China

**Keywords:** sepsis, pathogenesis, biomarkers, therapeutic drugs

## Abstract

Sepsis is defined as “a life-threatening organ dysfunction caused by a host’s dysfunctional response to infection”. Although the treatment of sepsis has developed rapidly in the past few years, sepsis incidence and mortality in clinical treatment is still climbing. Moreover, because of the diverse manifestations of sepsis, clinicians continue to face severe challenges in the diagnosis, treatment, and management of patients with sepsis. Here, we review the recent development in our understanding regarding the cellular pathogenesis and the target of clinical diagnosis of sepsis, with the goal of enhancing the current understanding of sepsis. The present state of research on targeted therapeutic drugs is also elaborated upon to provide information for the treatment of sepsis.

Sepsis affects more than 30 million people annually worldwide, and is one of the major causes of death in critical patients worldwide. Any infected person can potentially develop sepsis, and the incidence of sepsis is as high as 1–2% of all hospitalized patients. Therefore, the cost of treatment for sepsis is also the highest among all disease treatments [1]. In 2013, the total cost is about $23.6 billion for nearly 1.3 million inpatients affected by sepsis in USA, and this has increased at a yearly rate of 11.5% [2]. Although considerable progress has been made in medical technologies such as anti-infective treatments in recent years, the incidence of sepsis continues to rise. Moreover, even after treatment, patients with sepsis face long-term and serious problems in the form of physical, psychological, and cognitive disorders [3,4]. In 2012, the Global Sepsis Alliance and its founding members (including WFSICCM, WFPICCS, WFCCN, ISF, and SA) established World Sepsis Day on September 13 to enhance public awareness of this dangerous disease [5]. Therefore, the identification of effective methods for sepsis prevention, diagnosis, and treatment is an urgent need for human health.

The aim here is to review the pathophysiology of sepsis and provide the current research advances on the prevention, diagnosis, and treatment of this common disease.

## 1. Definition

Sepsis is defined as a syndrome involving physiological, pathological, and biochemical abnormalities caused by infection. In the fourth century B.C., Hippocrates first proposed the term “σήψις” (sepsis), with sepsis being regarded as a process of decay or decomposition of organic matter [6,7]. Aulus Cornelius Celsus and Galen described the characteristics of inflammation as redness, swelling, fever, pain, and loss of function [6,8]. In 1914, Schottmueller discovered that sepsis was a type of host system response elicited by pathogenic microorganisms that underwent blood circulation and caused excessive systemic inflammation [9]. Over the past few decades, numerous medical studies have presented definitions for sepsis, including those for septicemia, sepsis, toxemia, bacteremia, and endotoxemia [10]. To strengthen the clinician’s understanding of sepsis, to identify and diagnose sepsis at an early stage, and to effectively treat the disease, the international academic community has revised and updated the definition and diagnosis of sepsis thrice. The third definition of sepsis is “a life-threatening organ dysfunction caused by a host’s dysfunctional response to infection (sepsis-3)” was unanimously passed by the 45^th^ Society of Critical Care Medicine [11]. The new definition states that sepsis refers to the host’s uncontrolled response to infection and life-threatening organ dysfunction. This definition emphasizes the mechanism and severity of infection-induced organ dysfunction and requires timely identification and intervention in clinical treatment. Sepsis’ new diagnostic criteria differ for intensive care unit (ICU) and non-ICU patients. For patients with ICU infection or suspected infection, when the Sequential Organ Failure Assessment (SOFA) score is 2 points, the diagnosis is sepsis; for patients with a non-ICU infection or suspected infection, there are two quick Sequential Organ Failure Assessment (qSOFA) scores (systolic blood pressure less than 100 mm Hg, respiratory rate loss greater than 22 times/min, a change in consciousness) or more than two positive diagnoses as sepsis [12,13,14].

According to this new definition, the importance of the infection-induced unsteady host response was emphasized, which extends beyond the possibility of death directly from the infection itself and emphasizes the requirement for timely diagnosis. Concurrently, the description of sepsis 3.0 means that the concept of “severe sepsis” no longer exists, and the definition of septic shock has been updated to include the severe circulatory disorders and cellular metabolic disorders caused by sepsis, as well as the markedly higher risk of death than that from sepsis alone [10,15]. A clinical characteristic of septic shock also includes patients with septic shock who can be clinically identified by a vasopressor requirement to maintain a mean arterial pressure of 65 mm Hg or greater, and the serum lactate level greater than 2 mmol/L (>18 mg/dL) in the absence of hypovolemia [10]. 

However, according to the latest definition of sepsis, the accuracy of clinically implemented diagnostic criteria including Sequential Organ Failure Assessment (SOFA), Systemic Inflammatory Response Syndrome (SIRS), Logistic Organ Dysfunction Score, and quick Sequential Organ Failure Assessment (qSOFA) are ambiguous. Therefore, a measurement has been developed by Dr. Seymour of the Department of Critical Care Medicine, University of Pittsburgh School of Medicine, based upon the multicenter clinical data collection, suggesting that the predictive validity of qSOFA for inpatient mortality was higher than that of SOFA and SIRS among patients suspected of infection [10]. The qSOFA standard can be used to encourage clinicians to further study organ dysfunction, initiate or escalate treatment where appropriate, and consider referral to intensive care or the increase in the frequency of monitoring when needed [16]. However, the new diagnostic criteria are derived from complex retrospective studies, so Sepsis 3.0 was questioned and challenged as soon as it was published. To begin with, the use of qSOFA and SOFA standards was also warned by the American College of Chest Physicians, since it might lead to delayed diagnosis of severe infections and delays in treatment [17]. It is suggested that even if the scores of qSOFA or SOFA do not match 2 or more, the treatment of infection and the necessary care should not be postponed. In addition, since the qSOFA presentation, the diagnostic criterion has been controversial. The qSOFA score is derived from the retrospective analysis of non-ICU patients. It is difficult to collect the clinical information of patients outside the ICU. It may lead to the lack of a comprehensive and objective data information base for the development of qSOFA scores. The current qSOFA score may be too sensitive and specific. Insufficient sex, such as patients with a basic blood pressure below 100 mmHg, pain, irritability, etc., resulting in a respiratory rate of 22 times/min to reach the qSOFA standard, may lead to an overdiagnosis of sepsis. In other words, the qSOFA maybe oversensitive and exhibits a low specificity, which may lead to an overdiagnosis of sepsis; therefore, the qSOFA predictive value is suggested to be lower than other commonly used scoring systems [14,17,18]. Thus, neither qSOFA nor SOFA or SIRS is an independent definition of sepsis. Nevertheless, these measurements should be conjunctively used in managing sepsis patients [19].

## 2. Pathogenesis of Sepsis

**Figure 1:**
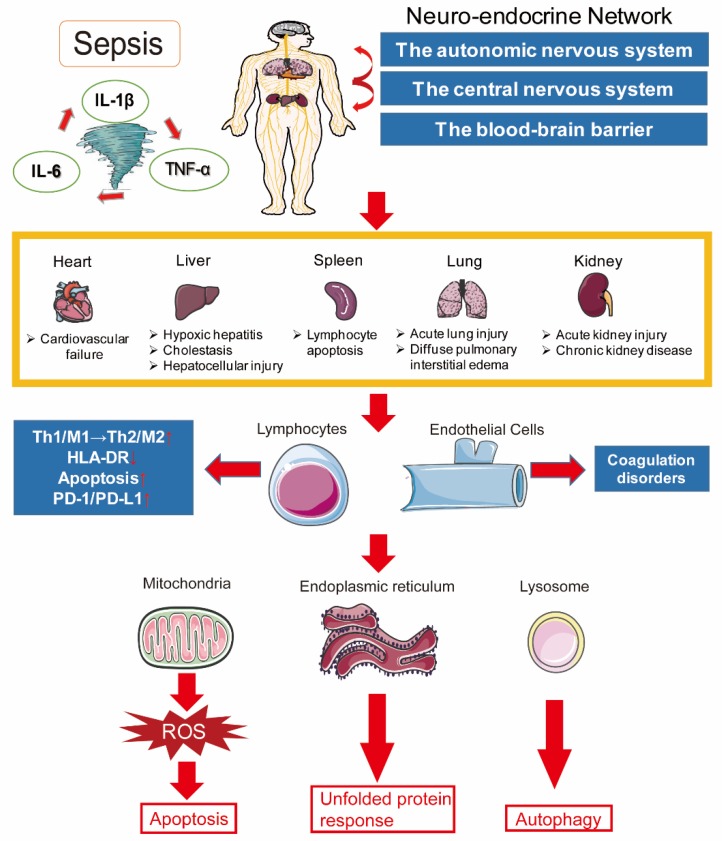
The complex pathogenesis of sepsis.

Sepsis is not only a process of systemic inflammatory response or immune disorder, it rather involves changes in the function of multiple organs in the body. As depicted in Figure 1, on the cellular and molecular levels, the pathogenesis of sepsis is extremely complex, including imbalance in inflammatory response, immune dysfunction, mitochondrial damage, coagulopathy, neuroendocrine immune network abnormalities, endoplasmic reticulum stress, autophagy, and other pathophysiological processes, and ultimately leads to organ dysfunction, which will be dissected in the following.

### 2.1. Inflammation Imbalance

Inflammatory imbalance represents the most critical basis of sepsis pathogenesis and occurs throughout the whole process of sepsis, and the pathogens eliciting the response include organisms such as bacteria, fungi, parasites, and viruses. The host’s initial acute response to invasive pathogens typically causes macrophages to engulf the pathogens and produce a range of pro-inflammatory cytokines, and this can trigger cytokine storms and activate the innate immune system [20,21]. Obviously, the activation of the innate immune system is mediated by pattern-recognition receptors (PRRs), which initiate a series of activation in immune cells by detecting damage-associated molecular patterns (DAMPs) or pathogen-associated molecular patterns (PAMPs), and thus upregulate the expression of inflammation-related genes [22]. In the immune response to sepsis, both exogenous factors derived from the pathogen (e.g., lipopolysaccharide (LPS)) and endogenous factors released by injured cells (e.g., high-mobility group box-1 (HMGB-1) protein) can interact with various PRRs, such as Toll-like receptors (TLRs), C-type lectin receptors (CLRs), RIG-I like receptors (RLRs), and NOD-like receptors (NLRs) [22,23]. Among these receptors, TLRs have been most widely studied. The interaction between TLRs and their ligands is induced by their TIR domains, which leads to the activation of c-Jun N-terminal kinase (JNK), extracellular signal-regulated kinase 1/2 (ERK1/2), p38 mitogen-activated protein kinase (MAPK), and nuclear factor-κB (NF-κB) signaling pathways through the myeloid differentiation factor 88-dependent pathway. These events are followed by the production of inflammatory cytokines such as interleukin (IL)-1, IL-6, tumor necrosis factor-α (TNF-α), interferon (IFN) regulatory factor 7 (IRF7), and adaptor protein 1 (AP-1) [24]. These cascades of events of multiple signaling pathways are tightly controlled processes that involve many cytoplasmic and membrane-bound proteins, such as interleukin-1 receptor-associated kinase-M (IRAK-M), toll interacting protein (TOLLIP), suppressor of cytokine signaling 1 (SOCS1), single Ig IL-1R-related molecule (SIGIRR), growth Stimulation expressed gene 2 (ST2), and so on [25]. Moreover, TLR signaling is also regulated by a tight control of TLR expression on the cell membrane. Thereby, TLR4 and TLR2 mRNAs are highly expressed in patients with sepsis [26].

In addition, soluble cytosolic PRRs such as NLRs are also involved in sepsis-induced immune imbalance. NLRs contain the nucleotide-binding oligomeric domain (NOD) and LRR domain (similar to TLRs) [27]. The activation of some NLRs is regulated by the adaptor protein receptor-interacting protein kinase 2 (RIP2) (also known as RICK) leading to the activation of NF-κB and AP-1, while some other NLRs (such as NLRP and NLRC4) participate in the formation of protein complexes referring to the inflammasome [28]. The inflammasome cleaves the caspase-1 precursor into active caspase-1, and activated caspase-1 cleaves IL-1β and IL-18 precursors to release the cytokines IL-1β and IL-18 [29].

Thirdly, the CLR family includes dectins, DC-SIGN, and mannose-binding lectins. Dectins induce reactive oxygen species (ROS) production and activates inflammatory responses through Src and Syk kinases, whereas DC-SIGN participates in the recognition of *Leishmania*, viruses, and fungi, which acts through Raf-1-mediated signaling pathway; however, the negative regulation of CLR responses require further study [30]. 

Interestingly, in addition to the above-mentioned PPRs, the receptors for double-stranded RNA including RIG-I, MDA5, and LGP2 have been also found to be involved in sepsis-induced immune dysfunction [31,32,33,34,35].

PRRs can be activated by exogenous PAMPs and endogenous DAMPs. In the case of endogenous sepsis, liver cells are reported to release a large amount of HMGB-1, which binds to bacterial endotoxin (LPS); the bacterial endotoxin is transported to the cytoplasm through RAGE receptors expressed on vascular endothelial cells and macrophages, and this leads to cysteinase caspase-11-mediated cell death (pyroptosis) and results in shock, multiple organ failure, and death [36,37].

### 2.2. Immune Dysfunction

The pathogenesis of sepsis includes a decrease in HLA-DR, lymphocyte replication, programmed cell death/apoptosis induction, anti-inflammatory molecules expression increasing, and cell-associated co-suppressor receptors and ligands upregulation [38,39]. 

When inflammation occurs during sepsis, neutrophils interact with endothelial cells and migrate, driven by chemokines, to the inflammation site, where they recognize and phagocytose pathogens, release various active factors and proteolytic enzymes, and eliminate pathogens [40]. Mononuclear/macrophage cells are activated when stimulated by cytokines (e.g., granulocyte-macrophage colony-stimulating factor (GM-CSF), TNF-α, INF-γ) or by pathogenic microorganisms, chemical mediators, immune complexes, etc., and the activated cells phagocytose and kill multiple pathogens and present antigens^30^. Differentiated effector T cells further promote the activation of macrophages and secrete a large amount of active medium to cause damage and fibrosis of the tissues [41]. During sepsis, the maturation process of dendritic cells (DCs) of the spleen and lymph nodes has been impeded during sepsis [42]. During sepsis, DC activation also causes a rapid accumulation of innate immune cells (including monocytes, natural killer (NK) cells, and granulocytes). Monocytes play an important role in the pathophysiology of sepsis. In the sepsis patients, the defects of monocyte metabolism are an expression of immunosuppression, which is characterized by an extensive inhibition of metabolic processes such as glycolysis, fatty acid oxidation, and oxidative phosphorylation [43]. NK cells can accumulate to produce elevated levels of INF-γ, but lost the ability to support the Th1 immune response required for the clearance of bacterial infection [44]. Although a large portion of sepsis patients might die during the initial cytokine storm, patients who survive this stage could develop immunosuppression, which includes failure to clear primary infections, the development of secondary opportunistic infections, and the reactivation of potential viruses. Sepsis-induced immunosuppression involves both innate and adaptive immunity. Immunosuppression after sepsis has been described as compensatory anti-inflammatory response syndrome^27^, and is regulated by co-stimulatory molecules such as CD80/B7-1, which are produced by activation of the TLR signaling pathway, and the naive T cells transformed into regulatory T cells induced by cytokines, and this results in a reduced expression of antigen presentation-related transcription factors (e.g., IRF4, MUM1) [45,46,47].

### 2.3. Mitochondrial Damage

Mitochondria are the major micro-organelles involved in energy production, protein synthesis, and catabolism. However, sepsis-induced mitochondrial damage or dysfunction can result in cellular metabolic disorders, insufficient energy production, and oxidative stress, which give rise to the apoptosis of organ cells and immune cells, thus ultimately generate immune disorders, multiple organ failure, and even death. 

**Figure 2:**
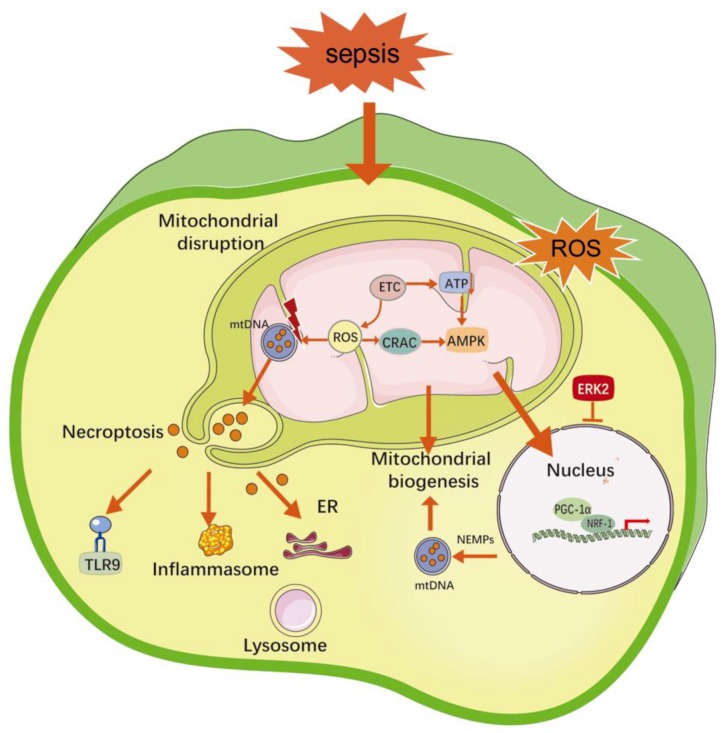
The regulation mechanisms of mitochondrial damage during sepsis.

As depicted in Figure 2, during sepsis, on account of a limited oxygen supply and incomplete oxidative reaction, as well as hypoxia, the free radical production increases dramatically while the machinery of the antioxidant system becomes damaged [48]. When exposed to DAMPs or PAMPs, activated leukocytes release inflammatory cytokines, which trigger the expression of NADPH oxidase [49]. As shown in the Figure 2, cytokines cause an overproduction of reactive nitrogen species (RNS) and NO by promoting inducible nitric oxide synthase (iNOS) activity. NO can bind to ROS peroxides to form RNS, and this leads to an irreversible inhibition of electron transfer chain (ETC) activity. Given this dysfunction of the ETC, mitochondria themselves represent a source of additional ROS production during sepsis, which unfortunately brings about further damage to mitochondria, including intimal damage, an inhibition of ETC activity, and mitochondrial DNA damage [50]. Ultimately, the mitochondrial matrix swells, the mitochondrial membrane ruptures, and apoptosis is initiated. A high rate of apoptosis takes place among splenic lymphocytes and among cells of other organs during sepsis, and an inhibition of apoptosis by using caspase inhibitors has been found to increase the survival rate in sepsis [51]. During LPS-induced sepsis, the nuclear respiratory factor-1 (NRF-1), which is a transcriptional activator of mitochondrial transcription factor A (TFAM), is upregulated in hepatocytes [52]. Autophagy is activated in response to the clearance of irreversibly damaged mitochondria. Mitochondrial biogenesis is regulated by the AMPK/PGC-1α/NRF-1/2 signaling pathway; nevertheless, the disproportionality of the ATP/ADP ratio as a result of insufficient ATP production disrupts the activation of AMPK and the subsequent PGC-1α/NRF-1/2 pathway and thereby contributes to TFAM expression [53,54]. As a transcription promoter, TFAM translocates into the mitochondrial matrix and causes mitochondrial DNA expression after mitochondrial biogenesis. It appears that the mitochondrial density continues to decline after a severe sepsis episode [55]. More experimental investigations remain to be launched for the role of the mitochondrial control mechanisms especially in inducing the multiple organ failure that is characteristic of sepsis and as potential therapeutic targets [56,57,58].

### 2.4. Coagulation Disorders

The interaction between inflammation and coagulation is widely considered to be a key point in the pathogenesis of sepsis. Inflammation can induce a coagulation reaction in sepsis, and activation of the coagulation reaction promotes the inflammatory response [59].

Under normal conditions, the activation of coagulation is regulated by three critical physiological anticoagulant pathway systems, including the tissue factor pathway inhibitor system, the activated protein C (APC) system, and the antithrombotic system, which regulated the activation of coagulation [60]. During sepsis, all three pathways exhibit a certain degree of disorder. Due to impaired protein synthesis, the levels of sustained consumption and protein degradation in the three coagulation-inhibitor pathways are low. Thrombomodulin (TM) and endothelial protein C receptor expression is downregulated due to the conversion of protein C to APC under inflammatory conditions [61]. Furthermore, during maximal activation of coagulation, the endogenous fibrinolysis diminishes substantially in sepsis; when plasminogen activator (i.e., tissue plasminogen activator (t-PA)) and urokinase-type plasminogen activator (u-PA) are released from vascular endothelial cell storage sites, plasminogen activator stimulation and sub-quantitative plasmin production increase, whereas a continued increase in plasminogen activator inhibitor-1 (PAI-1) makes this effect disappear [62]. The PAI-1 polymorphism has been shown to increase the risk of septic shock caused by meningococcal infection. Patients featured with the 4G/4G genotype present highly elevated concentrations of PAI-1 and increased mortality related to clinical outcome in Gram-negative sepsis [63]. 

### 2.5. Neuroendocrine–Immune Network Abnormalities

The homeostasis that depends on the interaction of the neuroendocrine–immune system is also considered to be a crucial part of the host response during septic shock [64,65]. In the case of threats, the central nervous system responds to sepsis through three main mechanisms: (1) the autonomic nervous system, in which primary afferent nerves (vagus and trigeminal nerves) and sensory nerves are associated with PAMPs and lead to inflammatory cytokine activation; (2) circulatory inflammatory mediators, via the choroid plexus and the ventricle organs connected to the central nervous system; and (3) by means of activation of endothelial cells through the blood–brain barrier, causing the release inflammatory mediators (NOS metabolites) [66].

The immune system can be regarded as a “diffuse sensory organ” that signals to the brain through distinct pathways, such as through the vagus nerve and endothelial activation/dysfunction, and this results in the liberation of cytokines and neurotoxic mediators. These afferent signals trigger the efferent response of the central nervous system and thereby activate the autonomic nervous system (including the sympathetic nervous systems and parasympathetic nervous systems), associated with the hypothalamic–pituitary–adrenal (HPA) axis activation, which is the most crucial part. Under normal conditions, the hypothalamic paraventricular nucleus and the supraoptic nucleus release corticotropin (CRH) and arginine vasopressin (AVP). In the pituitary gland, AVP can enhance CRH release. Subsequently, AVP and CRH stimulate the release of adrenocorticotrophic hormone (ACTH), which, in turn, is responsible for the secretion of adrenal cortisol, which counteracts the inflammatory process and restores cardiovascular homeostasis. HPA axis dysfunction during sepsis reduces serum levels of CRH, ACTH, and adrenal cortisol, leading to adrenal insufficiency syndrome [67]. Moreover, a branch of the autonomic nervous system, mainly the sympathetic branch, regulates cytokine production [68]. Evidence indicates that noradrenaline (NA) can respond to LPS; NA is released from the non-synaptic ends of sympathetic axons, is detected in increased concentrations near immune cells, and can inhibit the expression of the pro-inflammatory factors TNF-α and IL-12 and promote expression of the anti-inflammatory cytokine IL-10 [69,70]. These results support the idea that this effect is mediated by β2-adrenergic receptors, which are expressed on immune cells and coupled to cAMP [71].

**Figure 3:**
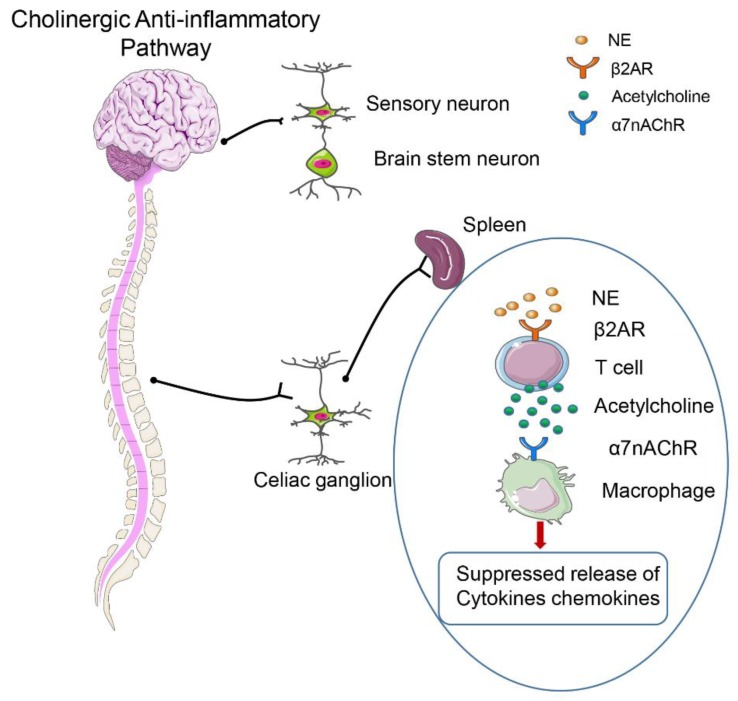
The function of the cholinergic anti-inflammatory pathway (CAP) in sepsis.

As shown in Figure 3, the neurotransmitter acetylcholine (ACh) signaling plays an important role in modulating inflammatory responses. The concept of the “cholinergic anti-inflammatory pathway (CAP)” has been proposed in a study that examines how the vagus nerve participates in the regulation of sepsis [72], which opens up a new direction for the treatment of sepsis. The CAP activation can lead to inhibition of the synthesis and release of cytokines, and the differentiation and maturation of T cells followed by a substantial reduction of the killing function of monocytes and neutrophils [73]. CAP is currently considered to exert anti-inflammatory effects mainly through the vagus nerve, Ach, and its specific α7 nicotinic ACh receptor (α7 nAChR), and related intracellular signal transduction pathways. Upon interaction with ACh, α7 nAChR causes a decrease in the level of pro-inflammatory factors, reduces the expression of chemokines and adhesion molecules, alters the differentiation and activation of immune cells, and regulates homeostasis [72]; thus, it acts as an “effector” to exert anti-inflammatory effects in CAP. After the vagus nerve is severed, the susceptibility to endotoxic shock is increased [74,75]. Therefore, systemic inflammatory response can be alleviated by stimulating the efferent vagus nerve or activating α7 nAChR. In several in vitro cellular models, the α7 nAChR also plays a key role in anti-inflammatory effects via the activation of diverse signaling pathways. Therefore, α7 nAChR is considered to be a target for regulating the release of inflammatory cytokines and the anti-inflammatory effects of CAP signaling.

### 2.6. Endoplasmic Reticulum Stress

The endoplasmic reticulum (ER) is an intracellular organelle that is involved in protein translocation, folding, posttranslational modification, and further transport to the Golgi apparatus. The unfolded or misfolded proteins are accumulated in the ER during sepsis, altering its homeostasis, and leading to oxidative stress and severe calcium disorders that result in ER stress [76]. Under ER stress, unfolded protein response sensors might switch their signals to stimulate the cell death by unique registration signaling mechanisms, which include several steps: the first is transcriptional activation of the CEBP homologous protein (CHOP) gene, mediated by PKR-like endoplasmic reticulum kinase (PERK), inositol-requiring enzyme 1 (IRE1), and activating transcription factor 6 (ATF6); the activation of the JNK pathway, mediated by IRE, followed by the subsequent activation of TNF receptor-associated factor 2 and apoptotic signal-regulated kinase 1; finally, the activation of caspase-12 associated and the activated caspase-12 migrates from the ER to the cytosol and then cleaves caspase-9, and finally activates caspase-3 [77,78,79]. In sepsis animal models, markers of increased ER stress (such as glucose-regulated protein 94 (GRP94), CHOP, and caspase-12) are detected in several organs including the heart and liver, as well as these markers are directly connected with the extent of organ dysfunction, which may be a major cause for sepsis-induced multiple organ failure [80]. ER stress brings about abnormal apoptosis in sepsis animals, suggesting that ER stress-mediated apoptosis represents a potential new target for clinical prevention and treatment for sepsis.

### 2.7. Autophagy

Autophagy refers to the natural process by which a cytoplasmic substance or pathogen is engulfed by the autophagosome, which is then fused with a lysosome to be degraded. Autophagy is a critical defense mechanism used by the host to resist external pathogens and dangerous signals, and plays a critical role in the induction and regulation of natural immune-cell inflammatory response, and is a key factor affecting sepsis development [81]. Autophagy exerts a protective effect in sepsis probably through the following mechanisms: pathogen clearance, the neutralization of microbial toxins, the regulation of cytokine release, the reduction of apoptosis oncotarget, and the promotion of antigen expression [82,83,84]. In a study that genetic ablates the autophagy protein ATG16L1gene, the endotoxin effects were enhanced, and the autophagy-deficient mice became more susceptible to LPS challenge, since the immune response is inhibited due to T-cell autophagy deficiency [85]. In a mouse model of sepsis developed using cecal ligation and puncture (CLP), incomplete autophagy might lead to cardiac dysfunction in sepsis, and autophagy activation by rapamycin restores cardiac function and reduces CLP-induced myocardial damage [19].

## 3. Biomarkers of Sepsis

Since sepsis is a serious disease characterized by stimulation of the systemic inflammatory response to infection, specific sepsis-related biomarkers should be utilized for clinical diagnosis for the rapid identification of infections, rational employment of antibiotics, evaluation of the affection of interventions, therapeutic monitoring of organ function for prognosis, and so on.

### 3.1. Infection-Related Biomarkers

#### 3.1.1. Procalcitonin (PCT)

PCT is a precursor of the hormone calcitonin secreted by C cells from the thyroid gland. To investigate the significance of dynamic procalcitonin (PCT) monitoring in the guiding of antibiotics for the sepsis patients in intensive care units (ICU), Pundiche et al. retrospectively analyzed the treatment of 73 patients with sepsis. The results suggest that a dynamic measurement of PCT may be a predictor for life-threatening antibiotic infection [86]. PCT is an acute-phase protein that is secreted by various tissues under endogenous and exogenous stimulation by molecules such as cytokines and LPS, and PCT is a chemokine for blood monocytes [87]. In severe bacterial infections or sepsis, PCT appears earlier than other inflammatory factors as a clinical diagnostic marker for sepsis. During sepsis, PCT expression increased significantly within 2 to 6 h and peaked at 6 to 24 h [88]. The prognostic performance of PCT was investigated in a meta-analysis of 25 studies, in which 2353 patients were admitted to the ICU, emergency department, or general ward; a statistically significant difference was observed in the mean PCT values between survivors (*n* = 1626) and non-survivors (*n* = 727) on Day 1 (*p* = 0.02), and the mean difference on Day 3 was also significant (*p* = 0.002) [89]. However, in a subgroup comprising patients with severe sepsis and septic shock, a significant difference was not found on Day 1 (*p* = 0.62) [89]. Based on the comprehensive analysis of all biological markers of inflammatory response, PCT is considered to be the best choice among the recommendations of the guidelines [90,91]. When PCT drops above 80% to 90% or PCT <0.5 ng/mL, a discontinuation of antibiotics can reduce bacterial resistance and does not affect the extension of hospital stays; the relative mortality is within 28 days of admission [92]. Therefore, the current reference indication for antibiotics withdrawal is when serum PCT <0.25 ng/mL [93]. Thus, PCT is used as one of the serum markers for the early diagnosis of sepsis or septic shock, and displays a higher sensitivity and specificity than traditional serum markers.

#### 3.1.2. C-Reactive Protein (CRP)

Known as C-reactive protein, CRP is an acute-phase protein synthesized by hepatocytes when the body is affected by microbial invasion or tissue damage. CRP is the most studied infection and inflammation marker [94]. To measure the ability of the clinical value of dynamic C-reaction protein (CRP) monitoring in the diagnosis of sepsis patients, a meta-analysis was performed with 495 patients in the sepsis group and 873 patients in the non-sepsis group to assess the diagnostic accuracy of C-reactive protein (CRP) for sepsis. The results indicate that the value of CRP for the diagnosis of sepsis patients is a moderate degree [95]. As compared with the levels of other acute-phase proteins, the CRP level increases substantially. During infection by pathogens, the CRP level increases within 6–8 h and peaks after 36–50 h [96]. As a marker of bacterial infection, the sensitivity and specificity of CRP have been reported to be 68–92% and 40–67%, respectively. Moreover, the CRP level within 48 h before treatment has been shown to potentially help in assessing the response of patients with sepsis to initiate antimicrobial therapy, and the CRP level at admission might serve as a useful marker of early infection. Subsequently, CRP levels begin to decline, and this might be as a result of the remission of inflammation and as well as response to antibiotic treatment in the clinical setting [97]. Nevertheless, CRP continues to contribute to the prognosis and treatment progress monitoring in the case of sepsis, and elevated CRP levels might have a relation to the extent of the disease and the severity of the infection [98]. 

#### 3.1.3. Cytokines (TNF-α/IL-6)

TNF-α is a pro-inflammatory cytokine that has been widely studied in the pathophysiology of sepsis. TNF-α helps activate endothelial cells to attract neutrophils. Within 30 min of infection, macrophages release TNF-α, which acts as a mediator and a regulator of the innate immune response [99]. Under the combined action of anti-inflammatory cytokines, a sustained increase in TNF-α levels leads to an aggravation of inflammation, and this also involves organ damage, which results in an increase in the mortality rate in patients with sepsis. Given this mechanism of action, TNF-α could serve as a critical prognostic marker for sepsis.

IL-6, a multi-directional cytokine, is a 21-kDa glycoprotein that is not only produced mainly by the macrophages and lymphocytes, but is also expressed by various cells in response to infection [100]. IL-6 can potentially affect the activation of B and T lymphocytes. In the case of burn patients or patients undergoing major surgery, an early rise (from 1 to 6 h) in IL-6 levels is displayed and found to be associated with several indicators of disease severity. Current clinical studies on post-surgery patients with severe sepsis show that IL-6 is markedly diminished in the first week of infection among survivors; however, among non-survivors, IL-6 increases during the first week of infection [101]. As a common life-threatening disease among infants, IL-6 measurement in neonatal sepsis (NS) diagnosing has been shown to serve as an effective, noninvasive, and rapid method. For example, in a study that measured the effectiveness of using IL-6 in predicting NS and included both 353 infants with sepsis and 691 normal infants, the results demonstrated that the sensitivity and specificity of IL-6 were 0.79 and 0.84, respectively. In addition, the maximal joint sensitivity and specificity (Q value) in the summary receiver operating characteristic curves was 0.82, and the area under the curve was 0.89 [102]. A meta-analysis showed that IL-6 exhibited a combined sensitivity of 80% for sepsis and a specificity of 85%, and in the neonatal group, the combined sensitivity was 77.0% and specificity was 91.0% [103]. Thus, IL-6 appears to be an effective indicator for predicting NS, and could be used as a reference for the early diagnosis of sepsis in neonatal ICUs. To determine the clinical diagnosis value of dynamic serum IL-1β, IL-6, IL-8, and TNF-α levels in neonatal sepsis, the prospective study was performed on hospitalizing neonatal sepsis who were classified as culture-proven sepsis (*n* = 12), as culture-negative sepsis (*n* = 21), and as healthy newborns (*n* = 17). The results indicate that the inflammatory mediators IL-1β, IL-6, IL-8, and TNF-α can be used to diagnose and assess the therapeutic efficacy of neonatal sepsis [104].

### 3.2. Biomarkers Related to Inflammation Activation and Immune Imbalance

#### 3.2.1. Monocyte Chemoattractant Protein-1 (MCP-1)

As a small cytokine, MCP-1 belongs to the CC chemokine family. To identify prognostic biomarkers for sepsis, a prospective cohort study was taken by using the chemokine/cytokine array and enzyme-linked immunosorbent assay. In total, 143 patients with sepsis were enrolled and divided into survivor (*n* = 87) and non-survivor groups (*n* = 56) according to the 28-day mortality status. As the result, the survivor groups exhibited significantly lower plasma concentrations of MCP-1 compared to non-surviving with significant difference, which indicate that MCP-1 is a useful biomarker for predicting sepsis outcomes [105]. When inflammation occurs, monocytes, macrophages, fibroblasts, and vascular endothelial cells secrete MCP-1 and exert specific chemotactic activation effects on monocytes/macrophages. In a study involving 89 children with acute bacterial infections, blood levels of chemokines were assessed using cell-counting bead arrays; the participants included patients with community-acquired pneumonia, sepsis, and bacterial abscesses [106]. In the study, the median plasma MCP-1 levels in all three cohorts were considerably lower than the levels in 20 healthy controls (24.9 pg/mL). In another study, baseline cytokine concentrations were measured using Luminex^TM^ analysis; the results showed that serum MCP-1 levels in healthy males and females were 62.8 and 55.4 pg/mL, respectively. In children with meningococcal sepsis, serum MCP-1 levels were positively correlated with SOFA scores (r = 0.68), and in adults who died of sepsis, serum MCP-1 levels were meaningfully higher than those in survivors [107,108].

#### 3.2.2. Programmed Death Receptor-1 and Programmed Death Ligand-1 (PD-1/PD-L1)

PD-1 is known to be widely expressed on activated T cells, NK cells, and B cells, and is also expressed in hematopoietic and non-hematopoietic cells [109,110,111,112]. The ability of PD-1 to suppress T-cell activation is dependent on the phosphorylation of the immunoreceptor tyrosine-based switch motif, which leads to the phosphorylation of downstream effector molecules playing a negative regulatory role, and inhibiting T cell proliferation and cytokine production [38]. In the bodies of patients who died of sepsis, spleen cells exhibit severe dysfunction and the lungs also show immunosuppressive effects, which is probably due to the self-programmed death of immune cells caused by the immunosuppression in sepsis [108]. The expression of inhibitory receptors on the surface of T-cells is upregulated due to chronic antigen stimulation, which also suggests the important immunosuppressive mechanism in sepsis [113]. The expression of PD-1 and PD-L1 has been reported to increase in T cells and monocytes of sepsis patients and animal models, respectively. Increasing numbers of studies have demonstrated that PD-1/PD-L1 blockade can be used to treat sepsis. To investigate the significance of dynamic PD-1-related molecules monitoring in evaluating the risk stratification and prognosis of septic patients, the 76 septic shock patients, 59 septic patients, and 29 healthy controls were enrolled to measure the PD-1 and PD-L1 expression on T cell and monocytes by flow cytometry. The results showed that only monocyte PD-L1 was associated with risk stratification and mortality in 3–4-day septic patients [114]. So far, the effects of PD-1/PD-L1 blockade on sepsis survival have only been tested in animal studies and need to be verified in human patients. Nevertheless, the PD-1/PD-L1 pathway plays a considerable role in sepsis-induced immunosuppression, and the blockade of this pathway may have a therapeutic value. 

#### 3.2.3. Soluble Triggering Receptor Expressed on Myeloid Cells-1 (sTREM-1)

sTREM-1 is mainly distributed on the surface of polymorphoonuclear cells and mature monocytes. When TLR2 or TLR4 bind to its ligand, TREM-1 is upregulated [115]. The upregulation of sTREM-1 initiates an intracellular cascade, which gives rise to the increasing expression of TNF-α, IL-8, and IL-1β, as well as increasing neutrophil degranulation. Concurrently, the expression of the anti-inflammatory cytokine IL-10 is downregulated. sTREM-1 is released into the serum by the mechanism of metalloproteinase shedding, and sTREM-1 binds to the ligand TREM-1 and thereby attenuates TREM-1-mediated inflammatory response. To determine the clinical diagnosis value of dynamic sTREM-1 in sepsis, 80 sepsis patients and 80 healthy controls were enrolled with sequencing TREM-1 genetic variation by PCR. The results indicate that the sTREM-1, APACHE II, and rs2234237 polymorphism are risk factors for prognosis by logistic regression. Meanwhile, the biomarkers of sepsis prognosis assessment are the dynamic changes of serum sTREM-1 and rs2234237 polymorphism [116]. As an indicator for sepsis diagnosis, TREM-1 displays a highly favorable diagnostic ability in the identification of *Shigella* infection and sepsis, with a combined sensitivity of 79% and specificity of 80% [117]. sTREM-1 levels were also dramatically higher in neonates with sepsis than in healthy newborns [118].

#### 3.2.4. Complement Pathway

The complement pathway is a pivotal pathway for the expansion of pro-inflammatory cytokine production and caspase-11-dependent cell death [119]. In bacterial infections and sepsis, complement proteins act on the surface of microorganisms by interacting with a complement protein 3 (C3) fragment, which plays an integral role in the process of phagocytosis of microorganisms. Activation of the complement cascade results in the release of pro-inflammatory peptides such as complement component 5a (C5a) [120]. Ample evidence indicates that complement proteins promote the inflammatory process in patients with sepsis, and that C5a might represent a critical marker. Both mouse model and clinical studies have found notable changes in C5a levels under sepsis conditions [121,122]. C5a measurement can be used in the diagnosis of autoimmune inflammation, but these measurements are still not widely employed in the case of patients with sepsis. In addition, recent studies have found that the Cpb1–C3–C3aR pathway can serve as a novel diagnostic markers and effective therapeutic target for early stage sepsis; carboxypeptidase b1 (Cpb1) has been identified as a novel mediator of caspase-11 expression in macrophages by using CRISPR–Cas9-mediated genome-wide screening, and Cpb1 has been shown to mediate subsequent caspase-11-dependent cell death [123]. Cpb1 binds to and activates C3aR and thus regulates innate immune signaling, and caspase-11 expression amplifies MAPK activity and IFN-α-receptor activation downstream of TLR4 and influences disease severity in LPS-induced sepsis and endotoxemic mice.

#### 3.2.5. Neutrophil Surface Receptor (CD64)

Fc-γ receptor-1 (FcγR1), which commonly known as CD64, belongs to a family of immunoglobulins expressed mainly on macrophages and monocytes, and is also a key immunomodulator in innate and adaptive immune responses [124]. In healthy volunteers, FcγR1/CD64 is expressed at extremely low levels on neutrophils, but is significantly elevated after inflammation or infection. FcγR1/CD64 expression on neutrophils is also reported to be associated with the severity of SIRS and sepsis. The deletion of FcγR1 improves survival in septic mice by downregulating the TLR4 signaling pathway [125]. To investigate the clinical diagnosis value of CD64 expression in ill adult sepsis patients, 1986 patients were enrolled with the meta-analysis. The results showed that CD64 was a useful biomarker for the early diagnosis of sepsis patients [126]. Thus, FcγR1/CD64 is a potential target for inflammatory diseases, and its expression on neutrophils may be used to detect the presence of sepsis.

#### 3.2.6. MicroRNA (miRNA)

The involvement of miRNAs in the regulation of inflammatory responses has been widely studied [127]. To determine the clinical prognostic predictors value of dynamic serum miRNAs for sepsis patients, microarray screens were used to analyze the expression of serum miR-574-5p from 12 surviving and 12 non-surviving sepsis patients. The results showed that the miR-574-5p was better than any single indicator in prognostic predictors for the death of sepsis patients [128]. Many of these studies support the role of miRNAs in acting as an inhibitory effect on the inflammatory response, in particular, miR-9, miR-147, and miR-132^99^. In addition, miR-21 has been shown to inhibit the expression of pro-inflammatory mediator PDCD4 and NF-κB activation, and thereby restrain the inflammatory response [129]. The expression level of miR-21 was measured in LPS-stimulated NR8383 cells, and miR-21 was found to attenuate. In the other case, miRNA-218 expression is substantially attenuated in the lung tissue of rats with acute lung injury, whereas the expression of inflammatory cytokines is markedly increased, which might be related to the activation of RUNX2 and NF-κB [130]. Meanwhile, several investigations have reported that there are significant differences of white blood cells RNA transcripts such as CEACAM4, LAMP1, PLA2G7, and PLAC8 genes in the sepsis patients compared with a healthy crowd. It is more meaningful that the FAIM3:PLAC8 gene expression ratio is superior to the plasma PCT in the diagnosis of sepsis [39,131,132].

#### 3.2.7. Plasma Cell-Free DNA

Plasma cell-free DNA (cf-DNA) is a fragmented, small double-stranded molecule and cell-free extracellular DNA derived from cell necrosis or apoptosis, which is low in normal human plasma, and contains mainly mitochondrial DNA (mtDNA) and nuclear DNA (nDNA). In sepsis, mtDNA and nDNA are passively released into the circulation after cell lysis and necrosis; both mtDNA and nDNA bind to the recognition receptor (PRR), thereby inducing the production of inflammatory cytokines. It can also be used as a measure of the extent of shock and organ damage and to assess the prognosis of septic shock [133,134,135]. Studies have shown that ICU patients have higher cf-DNA concentrations than healthy ones. When the ICU patients progress to sepsis or death, their cf-DNA concentrations are higher than those derived from the other disease processes and survivors [136]. When the cut-off value of cf-DNA was 2.35 ng/μL, the sensitivity and specificity of sepsis were 88% and 94%, respectively [137]. Kung et al. determined plasma mtDNA levels in 68 consecutive patients with severe sepsis and 33 healthy patients as controls by real-time quantitative polymerase chain reaction. As a result, the plasma mtDNA concentration in the severe septic patients was significantly higher than that in healthy controls. Interestingly, the plasma mitochondrial DNA were significantly higher on days 1 and 4 in the non-survivor group, but similarly decreased over 7 days after treatment between the survivor and non-survivor groups. Meanwhile, the cut-off value is 198 ng/mL in the receiver operating characteristic (ROC) curve for plasma mitochondrial DNA levels on admission with the 91% sensitivity and 72% specificity. Moreover, only plasma mitochondrial DNA levels can be used independently to predict death, and the results show that plasma mitochondrial DNA concentration increases by 1.0 ng/mL and mortality increases by 0.7% [138].

#### 3.2.8. Presepsin (sCD14-ST) 

As a cell surface glycoprotein, cluster of differentiation 14 (CD14) exists in two forms, including the membrane by a glycosylphosphatidylinositol tail (mCD14) and a soluble form (sCD14). The level of sCD14 in the blood of healthy people is low; the concentration is only at a microgram level [139]. When mCD14 is detached from the cell membrane surface, the LPS-binding protein (LBP)–CD14 complex is released into the bloodstream to form sCD14, which plays an important role in immune regulation, including the transportation of other lipids, cell–cell interaction, and the recognition of apoptotic cells.

During inflammation, sCD14 is cleaved in the blood by proteases to form a 64-amino acid N-terminal fragment, which constitutes the sCD14 subtype (sCD14-ST), and is also known as presepsin [140,141]. Interestingly, there are an increasing number of studies illustrating the function of presepsin (sCD14-ST) in the diagnosis of sepsis and prediction of the severity and mortality of the disease [142,143,144]. The serum level of presepsin (sCD14-ST) in the healthy human body is very low when it is not infected, and it is almost undetectable, but the serum level is sharply increased when the microorganisms such as bacteria and fungal are infected [145,146]. Yaegashi et al. performed a comparative analysis of plasma presepsin concentrations in 75 healthy volunteers, 80 SIRS patients, and 66 patients with sepsis. As a result, the plasma presepsin concentration in patients with sepsis was significantly higher than that of the other two groups [147]. An increasing number of studies have demonstrated that presepsin (sCD14-ST) can significantly increase within 2 h and peak at 3 h after the onset of infection [148], and as a promising diagnostic biomarker for adult sepsis, many studies have shown that presepsin has advantages over PCT, CRP, and IL-6 in diagnosing the sensitivity and specificity of sepsis and assessing disease severity and prognosis [149,150,151,152]. In a rabbit model of experimental sepsis, presepsin was detected in the blood of the animals 2 h after surgery, peaked at 3 h, continued to increase for at least 5 h, and presepsin increased earlier than IL-6 and PCT [153]. In 100 critically ill patients, presepsin showed a comparable ability to predict sepsis as PCT [154]. Sozushima’s study showed that presepsin plasma concentrations gradually increased in sepsis, severe sepsis, and septic shock, supporting a positive correlation between presepsin and the severity of sepsis [155]. Several studies have shown that plasma concentration of presepsin is positively correlated with Sequential Organ Failure Assessment (SOFA) score and acute physiology and chronic health evaluation II (APACHE II) [155]. A retrospective study of 157 patients with sepsis confirmed the prognostic effect of presepsin in sepsis, and suggested that multiple marker controls may be beneficial for the optimal management of sepsis patients [156]. Therefore, sCD14-ST is closely related to sepsis, and is very promising as a candidate diagnostic indicator for sepsis.

### 3.3. Biomarkers Related to Organ Dysfunction

#### 3.3.1. Angiopoietin (Ang)

The Ang family is closely related to neovascularization. Bacterial endotoxin has been shown to regulate the Ang family, affect the function of vascular endothelial cells, cause vascular leakage, stimulate cells to migrate to surrounding tissues, trigger the activation of inflammation and coagulation pathways, and eventually cause organ dysfunction [157]. Therefore, maintaining the stability of endothelial cells is crucial for the treatment of sepsis. The vascular endothelial cell activation during sepsis involves the Ang–Tie system [158]. Ang-1 and Ang-2 induce the secretion of endothelial growth factor and play distinct roles in mediating vascular quiescence [159]. Ang-1 stabilizes endothelial cells and inhibits vascular leakage by activating the Tie-2 receptor; by comparison, Ang-2 disrupts the integrity of microvessels by blocking the Tie-2 receptor and thus causes vascular leakage, which is one of the main mechanisms of organ dysfunction [160]. In several clinical studies of sepsis, both a high level of Ang-2 and a low level of Ang-1 or high Ang-2/Ang-1 and low Ang-1/Tie-2 ratios have been found to be associated with poor clinical outcomes, organ dysfunction, and adverse outcomes in sepsis^61^ [161,162]; these studies also suggest that Ang-1 may have a protective function against organ dysfunction. To evaluate the clinical diagnosis value of dynamic Ang-2/Ang-1 ratios in sepsis, consecutive patients with sepsis (*n*  =  440) were enrolled, and 55 healthy blood donors were included as the control group. The results indicate that the Ang-2/Ang-1 ratios are valuable for risk stratification in sepsis patients [104].

#### 3.3.2. Matrix Metalloproteinases (MMPs)

MMPs and tissue inhibitors of metalloproteinases (TIMPs) are key mediators in the regulation of wound healing after internal injury [163]. Interestingly, in severe sepsis, the expression levels of MMP-9, TIMP-1, and TIMP-2 are also significantly elevated [164,165]. To investigate the MMP-2, MMP-9, TIMP-1, TIMP-2 and IL-6 plasma levels in patients with severe sepsis and to examine their association with prognosis, the 37 patients on day 1 of severe sepsis and 37 healthy volunteers were enrolled, and the protein levels were measured by ELISA methods. The results indicate that MMP-9, TIMP-2, and TIMP-1 showed the higher sensitivity, specificity, and positive predictive value for sepsis [165]. Furthermore, TIMP-1 has been confirmed to be a predictor of clinical outcomes in patients with severe sepsis, while TIMP-2 is regarded as an early biomarker for predicting acute kidney injury (AKI), which is a common complication of sepsis [166]. TIMP-2 expression is found to be strongly upregulated in mice exposed to CLP and HK-2 cells exposed to LPS [115]. In animal models, TIMP-2 expression in renal tissues is associated with severity of AKI in vivo, whereas TIMP-2 silencing improves CLP-induced pro-inflammatory cytokine release by inhibiting the NF-κB pathway, and TIMP-2 downregulation protects renal tissues against endotoxin-induced AKI. In cell models, TIMP-2 silencing alleviates LPS-induced cytokine release, apoptosis, and cell damage, with the inhibition of inflammatory cytokine release being primarily mediated by p-P65 [115,167]. These findings reveal the pathogenic role of TIMP-2 in AKI and suggest a new therapeutic target of AKI related to sepsis.

### 3.4. Challenge

Biomarkers play a major role in the early diagnosis of sepsis and risk stratification, guiding the use of antibiotics, severity, and prognosis, and the evaluation of efficacy [168]. More than 170 biomarkers have been identified for the assessment of sepsis, including PCT, CRP, TNF-α/IL-6, MCP-1, miRNA, and other indicators [169,170,171]. 

However, different biomarkers play different roles in the pathophysiological processes of sepsis. The misuse of certain biomarkers will lead to overdiagnosis and an excessive use of drugs such as antibiotics [172,173,174]. For example, due to the low sensitivity of pathogenic microbial blood cultures, an increasing number of molecular biodiagnostic fields rely on the detection of bacterial DNA in the blood to identify sepsis [175,176]. This new technique can cause an overdiagnosis of sepsis by misidentifying transient bacteremia without clinical significance and biological characteristics. As a result, life-saving measures for shock patients may overuse the broad-spectrum antibiotics in patients with mild sepsis. At the same time, there are still many important scientific issues that need to be resolved. For instance, what specific biomarkers are used to more effectively distinguish between systemic inflammatory responses caused by sepsis and other critical states? How to combine multiple biomarkers to further improve the accuracy of clinical diagnosis of sepsis? How to rationally select the time of use of antibiotics by monitoring the changes in biomarker levels in patients with confirmed sepsis, and avoid the abuse of antibiotics?

## 4. Specific Drugs for Treating Sepsis

With a deepening of the understanding of sepsis pathogenesis, research on the treatment of sepsis has also emerged. Antibiotics, antiviral drugs, vasoactive agents, etc. have been used for the traditional treatment of sepsis, but no specific and effective therapeutic drugs for clinical use are as yet available. Current drug development is mainly focused on regulating systemic inflammatory response, coagulopathy, and immune dysfunction, restoring the body’s pro-inflammatory and anti-inflammatory homeostasis, and improving patient prognosis.

### 4.1. Drugs Targeting Inflammatory Imbalance

In the early stages of sepsis, excessive inflammatory response and cytokine storms are the most critical factors driving the development of sepsis. Therefore, the timely and appropriate antagonism of excessive release of inflammatory mediators has emerged as one of the main targets in the development of drugs for treating sepsis.

#### 4.1.1. Cytokine Antagonists

During sepsis, the body releases large amounts of inflammatory factors, causing a spillover of inflammatory mediators. Early attempts to modulate immune responses in sepsis included an injection of antibodies that neutralize TNF-α; with these antibodies, favorable results were obtained in animal studies, but most of the antibodies failed in clinical phase III trials [177,178,179]. However, after phase II/III clinical trials, two antibodies remained promising: Afelimomab and CytoFab [180]. Afelimomab is an F(ab’)_2_ fragment of a human TNF-α monoclonal antibody [181]. In patients with severe sepsis and elevated IL-6, afelimomab at a dose of 1 mg/kg every 8 h was found to significantly reduce the levels of circulating TNF-α and IL-6 for 3 consecutive days; concurrently, the treatment accelerated the regression of organ dysfunction and reduced the 28-day all-cause mortality rate by 5.8% [182]. CytoFab is a sterile affinity-purified preparation of a sheep IgG Fab fragment obtained from the blood of healthy sheep immunized with recombinant human TNF-α. In a double-blind, placebo-controlled, randomized clinical study, CytoFab was administered for 100 h at both loading and maintenance doses; 38 and 43 patients were assigned to placebo and CytoFab groups. CytoFab treatment had no effect on mortality; however, the number of ventilator-free days increased from a mean of 9.8 days in the placebo group to 15.6 days in the CytoFab group (*p* = 0.021), and the number of ICU-free days increased from a mean of 7.6 days in the placebo group to 12.6 days in the CytoFab group (*p* = 0.030). The circulating concentrations of TNF-α and IL-6 were markedly lower in CytoFab-treated patients than in placebo-treated patients, and TNF-α was also decreased in bronchoalveolar lavage fluid.

#### 4.1.2. PRR Antagonist

TLR4 is a signal transduction receptor for bacterial LPS, and TLR4 inhibition is considered to be critical for two reasons: firstly, circulating LPS levels increase in sepsis, even in infections caused by Gram-positive cocci; secondly, some of the endogenous molecules released by the host cell during sepsis are TLR4 sensitizers, such as heat shock protein 60, HMGB-1, and hyaluronic acid. Eritoran and TAK-242 (resatorvid) are potent inhibitors of TLR4 [183,184]. Eritoran was developed by Eisai, but it was stopped due to the failure of a clinical phase III trial [185]. As a cyclohexene derivative, TAK-242 (resatorvid) binds to the TIR domain of TLR4 selectively [186]. TAK-242 administration in mice with LPS-induced endotoxemia prolonged survival and alleviated organ dysfunction, and when used in combination with imipenem in a mouse model of CLP-induced sepsis, mortality in diseased mice was diminished, and cytokine production was inhibited [187]. The co-treatment was found to reduce organ dysfunction, as indicated by circulating levels of urea and aminotransferases, but tissue bacterial load was not affected, which suggests that TAK-242 (resatorvid) did not alter the ability of the host to clear bacteria [188].

#### 4.1.3. Pathogen-Associated Molecular Antagonists

LPS is a bacterial endotoxin that plays a critical role in the pathogenesis of sepsis [188].

As a cyclic cationic polypeptide antibiotic produced by *Paenibacillus polymyxa*, polymyxin B exhibits antibacterial activity against Gram-negative bacteria and can bind to and neutralize endotoxins [187]. A polymyxin B hemoperfusion (PMX-HP) system for endotoxin clearance has been established by using polymyxin B as an immobilized adsorbent, and the clinical efficacy of PMX-HP for patients with severe sepsis and septic shock has been determined in several studies [189,190]. According to the study of Nemoto et al., PMX-HP treatment improved overall patient survival significantly comparing with the controls (41% versus 11%; *p* = 0.002) [191]. An early application of PMX-HP was also associated with reduced mortality, improved hemodynamics, and enhanced lung oxygenation in a septic shock trial [192]. A meta-analysis supports these benefits [193]. However, contrasting results have been presented by two recent studies. Especially, a study by Iwagami et al. demonstrated that PMX-HP did not show any survival benefit for the treatment in patients with septic shock, while a randomized controlled trial showed no marked decrease in the mortality of patients with septic shock after PMX-HP treatment [194,195].

### 4.2. Drugs for Coagulopathy

During the pathogenesis of sepsis, the coagulation system and the inflammatory reaction cooperate with and promote each other to cause the coagulation–anticoagulation imbalance, which eventually leads to an uncontrolled coagulation cascade [196]. Therefore, inhibition of the abnormal coagulation reaction can affect the pathological process of inflammation and sepsis, and thus produce a certain therapeutic effect.

#### 4.2.1. Recombinant Human APC (rhAPC)

For the treatment of severe sepsis, rhAPC is currently the only approved drug. rhAPC was licensed after a successful study in a patient with severe sepsis, who featured an APACHE II score of >25 [197]. rhAPC exerts antiapoptotic, anti-inflammatory, and anticoagulant effects, and acts by regulating the inflammatory cycle of malignant coagulation activated by severe sepsis [198]. However, the post-marketing of the PROWESS-SHOCK clinical study failed to demonstrate a clinical role of rhAPC in the treatment of severe sepsis and septic shock, which eventually led to the withdrawal of the drug [199].

#### 4.2.2. Recombinant Human Soluble Thrombosis Regulators

TM is produced by endothelial cells, and TM binding eliminates the procoagulant activity of thrombin and activates the ability of thrombin to convert protein C to APC. Experimental studies conducted using recombinant soluble thrombomodulators (e.g., recombinant TM (rTM)) have shown that the effect on thrombin is mediated through the action of circulating HMGB-1 [200]. rhTM circulates in a soluble form, with its concentration reflecting the severity of coagulopathy and organ failure in sepsis [201]. In the treatment of disseminated intravascular coagulation (DIC), atherosclerosis, and acute respiratory distress syndrome, plasma is reduced to normal levels [202]. rhTM has been licensed in Japan for more than a decade for DIC management [203]. One study analyzed the rhTM treatment of DIC patients with sepsis in three hospitals in Japan from January 2006 to June 2011.^12^ Of the 162 patients with septic DIC, 68 received rhTM, and 94 received no treatment. The results showed a significant reduction in mortality after rhTM treatment (adjusted hazard ratio, 0.45; 95% confidential interval, 0.26–0.77; *p* = 0.013) [204]. After adjusting for disease severity, the rTM treatment group showed a notably higher beneficial effect as compared with the control group. In the case of another recombinant human soluble thrombomodulator, ART-123 developed by Artisan Pharma, a randomized phase II clinical study has been completed in patients with sepsis and DIC, and the data released showed that the ART-123 is a safe intervention for the patients with sepsis and suspected DIC [205].

#### 4.2.3. Pentoxifylline

Pentoxifylline is used as an anticoagulant in clinical treatment of surgical vascular disease. Pentoxifylline was tested in a small phase II clinical study of neonates with sepsis; 20 patients were assigned to the placebo group for 6 consecutive days, and 17 patients were assigned to the pentoxifylline group [206]. Pentoxifylline treatment was reported to reduce the incidence of DIC and multiple organ dysfunction syndrome.

### 4.3. Drugs Against Immune Function Inhibition

With the improvement in the treatment of sepsis, the number of patients with multiple organ failure in the ICU has decreased substantially, and persistent immune dysfunction has become the leading cause of death in patients with advanced sepsis. Therefore, overcoming immune cell apoptosis and immunosuppression has become a new focus in the field of drug treatment research for sepsis [113,207].

#### 4.3.1. Cytokines

In most patients with severe sepsis or septic shock, the host is immunosuppressed, and immune function is downregulated. This impaired immune response has been widely recommended to be reversed by the addition of the cytokines granulocyte colony-stimulating factor (G-CSF) and granulocyte-macrophage colony-stimulating factor (GM-CSF) (ref). One meta-analysis included 12 randomized clinical trials in which treatment with G-CSF or GM-CSF was compared to placebo treatment [206]. Although no difference in in-hospital mortality was found, the treatment considerably increased the rate of infection reversal. Regardless of the clinical outcome, 19 patients with severe sepsis were treated with GM-CSF, their HLA-DR expression in circulating mononuclear cells increased, and the release of pro-inflammatory cytokines was substantially improved when TLR2 or TLR4 ligand was administered^126^. In the 19 treated patients, immune system analysis indicated effective response reversal [208]. In a larger trial, 163 patients were assigned to the placebo group or treated with G-CSF. Whereas G-CSF did not affect in-hospital mortality, the treatment was associated with the development of acute coagulopathy and elevated troponin I [209]. 

#### 4.3.2. Co-Inhibiting Molecular Inhibitors

**Figure 4:**
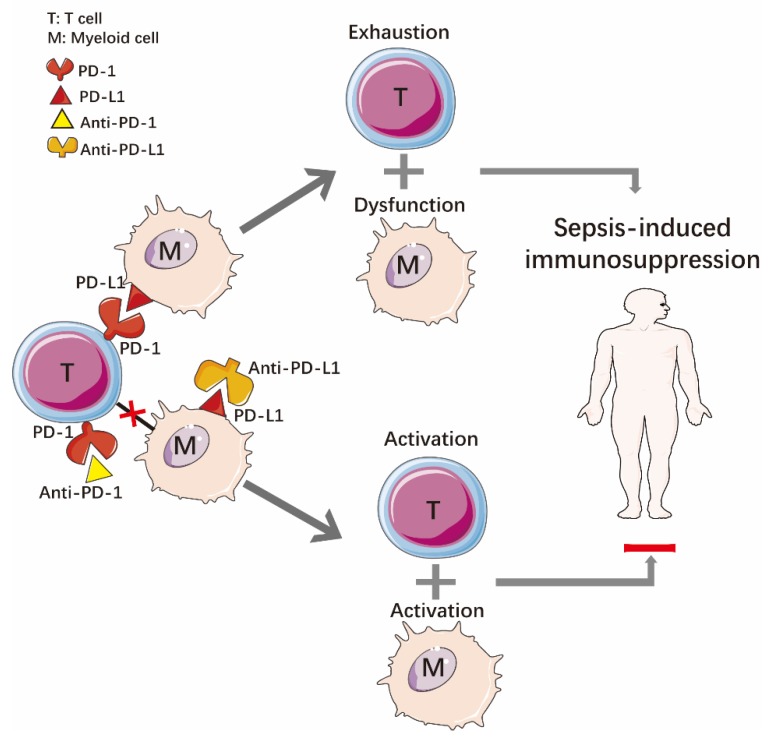
Blocking Programmed Death Receptor-1 and Programmed Death Ligand-1 (PD-1/PD-L1) signaling reverses the immunosuppression in sepsis.

Co-repressor molecules are considered to act as negative regulators of T-cell receptor-mediated T-cell activation and the cytokine-release processes in a class of acquired immune responses [210]. Negative costimulatory molecules have been shown to be involved in the development and progression of sepsis immunosuppression. PD-1, a negative costimulatory molecule, binds to its ligand, PD-L1, and transmits an inhibitory signal that blocks immune cell activation, proliferation, and effector function [211]. As depicted in Figure 4, in immunomodulatory therapy, blocking PD-1/PD-L1 signaling is one of the new methods to reverse the immunosuppression in sepsis [19,212]. Chang et al. confirmed the positive affection of anti-PD-1 and anti-PD-L1 antibodies on blood samples from 43 patients and 15 patients with sepsis and severe non-sepsis infection, respectively [110]. Meanwhile, anti-PD-1 or anti-PD-L1 antibodies reduced the level of apoptosis and increased the production of IFN-γ and IL-2 [110]. These findings support the conclusion that the reduction in PD-1/PD-L1 expression attenuated T-cell dysfunction and failure in patients with sepsis [110]. As expected, PD-L2 also plays an important grand scheme role in the innate immunity of sepsis [213]. Several studies have shown that targeting a checkpoint blockade could improve survival in sepsis, indicating that using co-inhibitory molecules is one of the most promising strategies for sepsis treatment in the future [214,215,216,217,218].

## 5. Future

Extensive research on sepsis has led to the identification of satisfactory prognostic markers that contribute to the diagnosis of sepsis. Recent studies on inflammation imbalance, immune dysfunction, mitochondrial damage, coagulation disorders, neuroendocrine–immune network abnormalities, endoplasmic reticulum stress, and autophagy have significantly advanced our understanding of pathogenesis of sepsis. However, we still lack a global perspective on cell levels, time, and space [219]; there is no full understanding of the pathogenesis of sepsis, and many questions remain. For instance, what is the mechanism for the initiation, maintenance, and termination of sepsis? What is the underlying mechanism of sepsis causing cellular and subcellular dysfunction? What the role of mitochondrial dysfunction in sepsis? How do the cytokines and co-inhibitory molecules co-regulate in the ill patients? What information can be used for the identification of organ dysfunction?

Traditional diagnostic practices are expensive and time consuming, and lack sufficient sensitivity and selectivity; thus, urgent demand exists for developing alternatives to available sepsis diagnostic systems. Although sepsis-related diagnosis biomarkers are evident in many studies, efficient and rapid diagnostic tools for the clinical diagnosis are still lacking. Combining patient clinical features with biomarkers for early diagnosis and risk assessment will be the future direction for the diagnosis of sepsis.

As sepsis is a systemic disease involving several organs, the pathogenesis of sepsis remains incompletely elucidated, and patients currently die even after admission. Current clinical guidelines can help implement effective management, restore adequate cell perfusion through early appropriate antibacterial therapy, and enable timely source control to improve the prognosis of patients with sepsis. 

Although many clinical trials are underway, there is currently no FDA-approved drug for the treatment of sepsis [168,220]. Moreover, additional focused research is required to strengthen our understanding of the basic pathophysiology and causes of death in sepsis. In the future, the classification of sepsis into distinct categories might help with identifying more direct and effective treatments for sepsis than those currently in use.

## Abbreviations

ACh	Acetylcholine
ACTH	Adrenocorticotrophic hormone
AKI	Acute kidney injury
Ang	Angiopoietin
AP-1	Adaptor protein 1
APC	Activated protein C
ATF6	Activating transcription factor 6
AVP	Arginine vasopressin
C3	Complement protein 3
C5a	Complement component 5a
CAP	Cholinergic anti-inflammatory pathway
CHOP	CEBP homologous protein
CLP	Cecal ligation and puncture
CLRs	C-type lectin receptors
Cpb1	Carboxypeptidase b1
CRP	C-reaction protein
DAMPs	Damage-associated molecular patterns
DCs	Dendritic cells
DIC	Disseminated intravascular coagulation
ER	Endoplasmic reticulum
ERK1/2	Extracellular signal-regulated kinase 1/2
ETC	Electron transfer chain
FcγR1	Fc-γ receptor-1
G-CSF	The cytokines granulocyte colony-stimulating factor
GM-CSF	Granulocyte-macrophage colony-stimulating factor
GRP94	Glucose-regulated protein 94
HMGB-1	High-mobility group box-1
HPA	Hypothalamic-pituitary-adrenal
IL-1	Interleukin-1
iNOS	Inducible nitric oxide synthase
IRAK-M	Interleukin-1 receptor-associated kinase-M
IRE1	Inositol-requiring enzyme 1
IRF7	Interferon regulatory factor 7
JNK	c-Jun N-terminal kinase
LODS	Logistic Organ Dysfunction Score
LPS	Lipopolysaccharide
MAPK	p38 mitogen-activated protein kinase
MCP-1	Monocyte chemoattractant protein-1
miRNA	MicroRNA
MMPs	Matrix metalloproteinases
NA	Noradrenaline
NF-κB	Nuclear factor-κB
NK cells	Natural killer cells
NLRs	NOD-like receptors
NRF-1	Nuclear respiratory factor-1
NS	Neonatal sepsis
PAI-1	Plasminogen activator inhibitor-1
PAMPs	Pathogen-associated molecular patterns
PCT	Procalcitonin
PD-1/PD-L1	Programmed death receptor-1 and programmed death ligand-1
PERK	PKR-like endoplasmic reticulum kinase
PMX-HP	Polymyxin B hemoperfusion
PRRs	Pattern-recognition receptors
qSOFA	Quick Sequential Organ Failure Assessment
rhAPC	Recombinant human APC
RLRs	RIG-I like receptors
RNS	Reactive nitrogen species
ROS	Reactive oxygen species
rTM	Recombinant TM
SIGIRR	Single Ig IL-1R-related molecule
SIRS	Systemic Inflammatory Response Syndrome
SOCS1	Suppressor of cytokine signaling 1
SOFA	Sequential Organ Failure Assessment
ST2	Stimulation expressed gene 2
sTREM-1	Soluble triggering receptor expressed on myeloid cells-1
TAK-242	Resatorvid
TFAM	Transcriptional activator of mitochondrial transcription factor A
TLRs	Toll-like receptors
TM	Thrombomodulin
TNF-α	Tumor necrosis factor-α
TOLLIP	Toll interacting protein
t-PA	Tissue plasminogen activator
u-PA	Urokinase-type plasminogen activator
α7nAChR	α7 nicotinic ACh receptor
